# Case Report: Durable response to mirvetuximab soravtansine in a patient with platinum-sensitive ovarian cancer and a diagnosis of myelodysplastic syndrome

**DOI:** 10.3389/fonc.2026.1898036

**Published:** 2026-07-09

**Authors:** Theresa Steindl-Tomschizek, Teresa Lucia Pan, Christian Sillaber, Edit Porpaczy, Zsuzsanna Bago-Horvath, Christoph Grimm, Nicole Concin, Stephan Polterauer

**Affiliations:** 1 Department of Obstetrics and Gynecology, Comprehensive Cancer Center Vienna, Division of General Gynecology and Gynecological Oncology, Medical University of Vienna, Vienna, Austria; 2 Department of Medicine I, Division of Hematology and Hemostaseology, Medical University of Vienna, Vienna, Austria; 3 Department of Pathology, Comprehensive Cancer Center, Medical University of Vienna, Vienna, Austria

**Keywords:** folate receptor alpha, mirvetuximab soravtansine, myelodysplastic syndrome, PSOC, thrombocytopenia

## Abstract

**Background:**

High-grade serous ovarian cancer is one of the most lethal malignancies in women, typically diagnosed at an advanced stage, treated with debulking surgery, platinum-based chemotherapy and maintenance therapy, and associated with a high risk of recurrence even after optimal treatment. Myelodysplastic syndrome is a rare but serious haematological disease which can occur after exposure to chemotherapy or PARP inhibitor therapy. Treatment of recurrent disease in patients who develop Myelodysplastic syndrome is particularly challenging due to the significant myelotoxicity associated with standard cytotoxic chemotherapy. Mirvetuximab soravtansine is an antibody-drug conjugate approved for use in platinum-resistant folate receptor alpha-positive high-grade ovarian cancer. Due to its limited myelotoxicity, it might be an interesting treatment option in patients with relevant haematologic side effects due to previous lines of chemotherapy.

**Case:**

We report on a patient with a recurrence of platinum-sensitive ovarian cancer (platinum-free interval: 16 months) who developed Myelodysplastic syndrome and ultimately, probably an acute Myeloid Leukaemia. Chemotherapy re-challenge with platinum-based combination chemotherapy was not feasible due to persistent thrombocytopenia (CTCAE grade 3). Therefore, treatment with mirvetuximab soravtansine was initiated showing durable response with a progression-free survival of nine months. The patient’s platelet count and tumor marker CA125 remained stable for nine months while she was receiving antibody-drug conjugate treatment.

**Summary and conclusion:**

Patients with underlying haematologic disorders sometimes cannot receive platinum-based chemotherapy due to its haematotoxicity, raising the need for alternative treatments for recurrent high-grade ovarian cancer. Data from clinical trials, supported by our case, suggest that mirvetuximab soravtansine may provide a promising option, allowing durable response without worsening pre-existing thrombocytopenia.

## Introduction

Ovarian cancer is a major contributor to the global cancer burden in women, with substantial incidence and mortality worldwide (1). High-grade serous ovarian carcinoma (HGSOC) is the most common histological subtype. As early disease is frequently asymptomatic and/or causes only non-specific symptoms, most patients are diagnosed at advanced stages (FIGO III–IV). Standard first-line treatment consists of cytoreductive surgery plus platinum-based chemotherapy, followed by maintenance therapy with a Poly(ADP-ribose)-Polymerase (PARP) inhibitor, bevacizumab, or their combination, selected according to BRCA/HRD status, residual disease, and prior bevacizumab exposure during chemotherapy.

Recurrent disease is traditionally considered platinum-sensitive (PSOC) if relapse occurs more than six months after the last platinum dose; in this situation, platinum-based combination chemotherapy is recommended. If recurrence occurs within 6 months, it is classified as platinum-resistant recurrence (PROC) and treatment with single-agent chemotherapy (plus bevacizumab) is recommended (2, 3).

Myelodysplastic syndromes (MDS) - termed myelodysplastic neoplasms in the revised 2022 WHO classification, with the two terms used synonymously herein - are clonal disorders arising from haematopoietic stem cells, characterized by ineffective haematopoiesis with bone marrow dysplasia and variable peripheral cytopenia. The estimated incidence in Europe is 1.8 per 100,000 individuals per year. In patients with advanced/recurrent ovarian cancer, the absolute risk of secondary MDS within five years was 0.3% after chemotherapy plus PARP inhibitor maintenance compared with 0.1% after chemotherapy alone. Clinically, MDS commonly manifests with pancytopenia (including severe neutropenia and thrombocytopenia), which is associated with increased susceptibility to infections, bleeding and high mortality (4, 5).

Given the thrombocytopenia associated with her MDS, platinum-based combination chemotherapy was not a feasible option for third-line treatment, prompting consideration of mirvetuximab soravtansine, an antibody-drug conjugate (ADC) that targets folate receptor alpha (FOLRα) consisting of an anti-FRα antibody linked via a cleavable disulfide bond to the tubulin-targeting agent maytansinoid DM4. The presence of FOLRα with moderate (2+) and/or strong (3+) intensity of membrane staining in >75% of tumor cells by immunohistochemistry must be confirmed prior to indication. Mirvetuximab soravtansine is approved as monotherapy in the treatment of PROC with strong biomarker expression (6).

This report describes the cause of death in a patient who developed MDS following surgery and systemic treatment for HGSOC with two lines of prior platinum-based chemotherapy, Bevacizumab and PARP inhibitor treatment. Due to her MDS with relevant thrombocytopenia, platinum-based combination chemotherapy was not an option. Therefore, the patient received mirvetuximab soravtansine as third-line therapy and showed durable response.

## Case presentation

**Figure 1:**
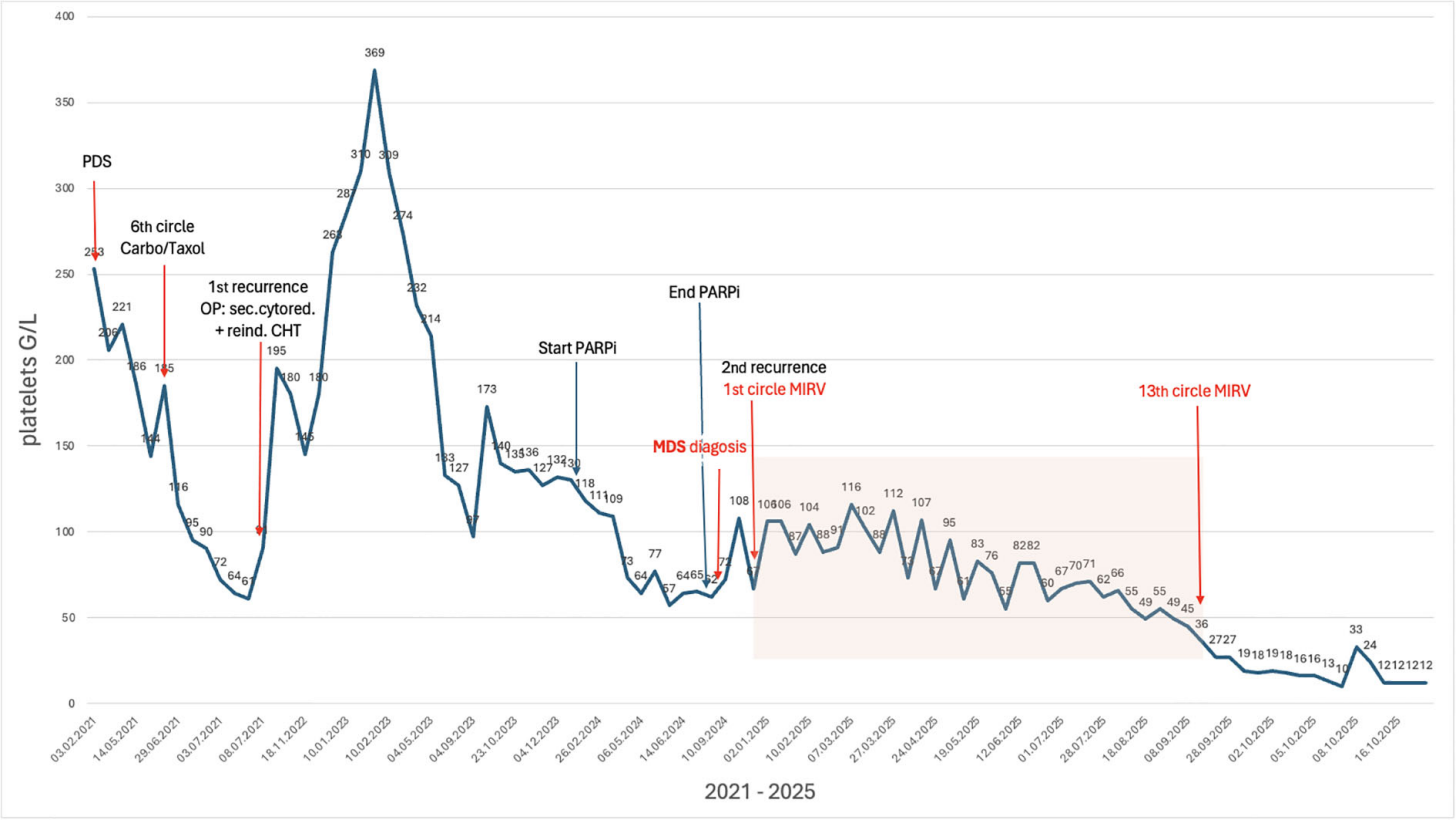
Course of platelet count during treatment and diagnosis of MDS. PDS, primary debulking surgery; OP, operation; reind. CHT, reinduction chemotherapy; MDS, myelodysplastic syndrome; MIRV, mirvetuximab soravtansine.

**Figure 2:**
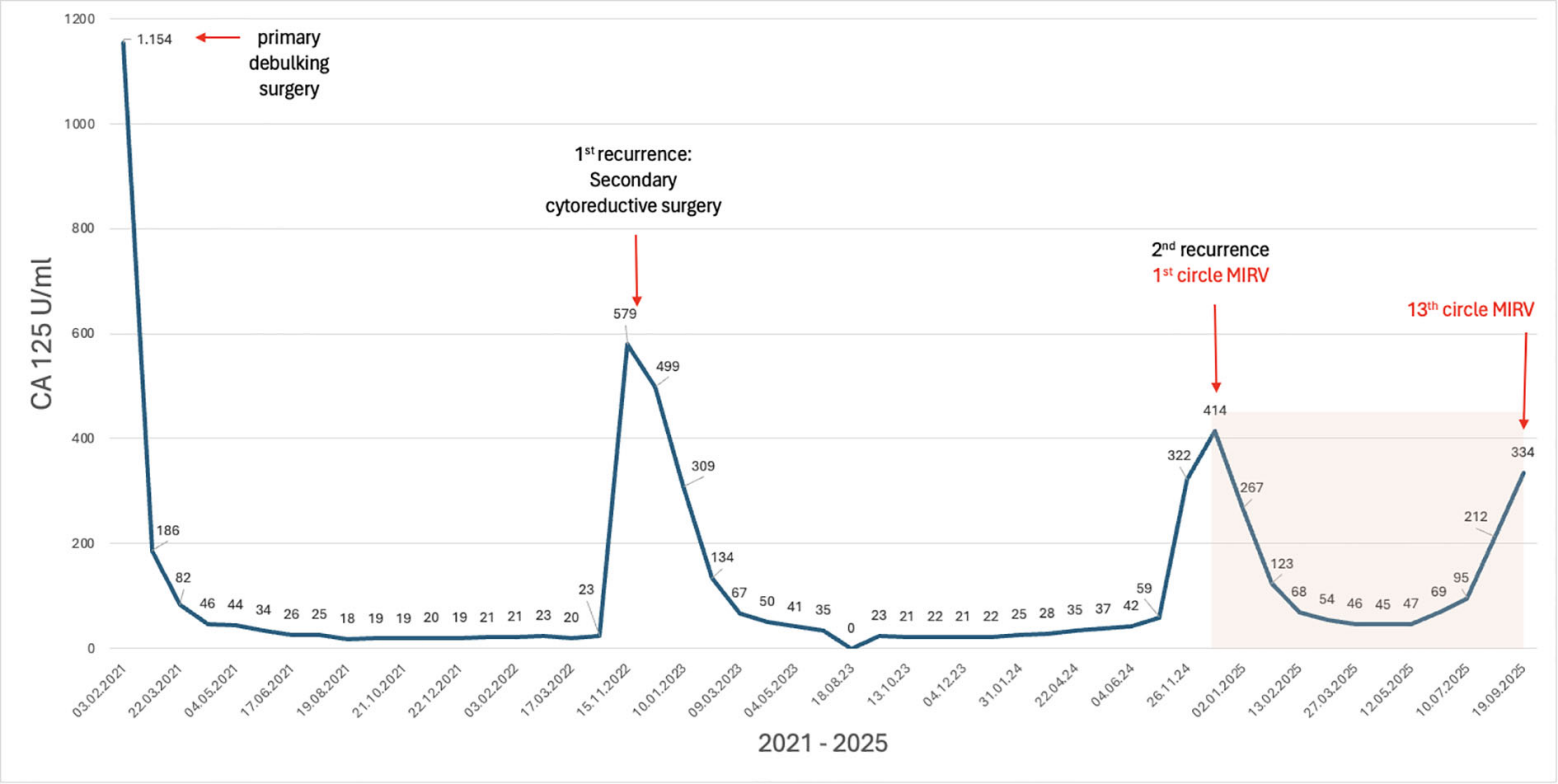
Course of CA 125 levels during treatment. MIRV, mirvetuximab soravtansine.

We report on a 51-year-old female patient with HGSOC FIGO stage IIIC. The patient had no relevant comorbidities, was not taking any regular medication, and reported a negative family history of malignancies. She underwent primary debulking surgery in February 2021. Molecular analysis revealed an HRD-negative tumor without detectable BRCA mutation (BRCA wild-type). The patient received adjuvant chemotherapy with six cycles of carboplatin and paclitaxel until June 2021, followed by a total of 19 cycles of bevacizumab until April 2022, which was discontinued due to disease progression. Eighteen months after the initial diagnosis, a recurrence was detected. A PET-CT scan revealed multifocal peritoneal carcinomatosis, as well as an FDG-avid lymph node at the level of the left aortic bifurcation. With preoperative suspicion of oligometastatic disease and positive DESKTOP criteria (ECOG 0, no ascites and R0 resection at primary debulking surgery) (7), the patient underwent secondary cytoreduction. However, intraoperatively, diffuse small bowel serosal carcinomatosis was detected and only a symptomatic large lymph node in the spleen area was removed (R1 resection). Afterwards, she received six cycles of carboplatin and pegylated liposomal doxorubicin until May 2023. Based on radiological and serological complete response, she received maintenance therapy with a PARP inhibitor for 12 months (July 2023 to July 2024). Due to recurrent thrombocytopenia, olaparib was initially dose-reduced. However, persistent thrombocytopenia despite dose modification ultimately led to permanent discontinuation of olaparib. In July 2024, a pelvic crest puncture was performed and the patient was diagnosed with a therapy-associated MDS. The biopsy revealed a TP53 mutation and a 5q deletion, as well as dysplastic changes in the bone marrow. These changes included ring sideroblasts, which were, however, only present sporadically. From this point onwards, the patient received continuous stimulatory factors and, later from August 2025, additional filgrastim once a week at a dose of 30, 000 IU. In October 2024, the patient presented with multifocal recurrence of her HGSOC (16 months platinum-free interval). Given her haematological disease, platinum-based combination chemotherapy was not an option. Treatment options were discussed and further immunohistochemical analysis of the primary tumor specimen demonstrated strong FOLRα positivity (>75% of tumor cells with moderate to strong [2+/3+] membranous staining intensity), meeting the biomarker threshold for treatment eligibility; the patient subsequently received a total of 13 cycles of mirvetuximab soravtansine starting from December 2024 until September 2025. During this period, the platelet count remained constant as can be seen in **Figure 1**. CA-125 serum levels showed partial response due to GCIG-criteria as can be seen in **Figure 2**. The patient reported mild keratopathy, which is a frequently detected side effect, and underwent treatment with hyaluronic acid eye drops. From the fifth cycle onwards therapeutic lenses were prescribed, which greatly improved her symptoms. It was only from the 10th cycle onwards that the patient began to complain increasingly about gastrointestinal side effects such as constipation, which was managed with laxative therapy. Due to moderate neutropenia (0.9 G/L) and thrombocytopenia (70 G/l), the patient received a reduced dose at the 9th cycle of the therapy. The first CT scan after four cycles of mirvetuximab soravtansine treatment showed partial remission according to RECIST including a significant regression of peritoneal carcinomatosis. Further imaging performed in July 2025 (after 10 cycles) showed stable disease. After a total of 13 cycles, the patient underwent CT scan in September 2025, which revealed progressive disease with increasing peritoneal carcinomatosis. CA-125 levels began to rise from the 9th cycle onwards. Continuous close communication with the patient was established before and during treatment with mirvetuximab soravtansine, and palliative care was offered, which she declined. After 13 cycles and a total of nine months of mirvetuximab soravtansine, the patient developed severe thrombocytopenia and febrile neutropenia, which resulted in a deterioration in her general condition. A suspected diagnosis was established that the MDS had transitioned into acute myeloid leukaemia (AML). Further investigation was not performed as the patient deceased at home in December 2025 due to general weakness and symptoms associated with her illness.

## Discussion and conclusion

Currently, the use of mirvetuximab soravtansine is limited to PROC tumors with high FOLRα expression. FOLRα can be detected in around 80% of HGSOC but is usually absent or severely limited in normal tissue. Encouraging results from the FORWARD II study in PSOC (overall response rate: 39%) led to the development of several subsequent trials, including the phase II studies PICCOLO, MIROVA, and Study-420, as well as the phase III GLORIOSA trial (8–12). PICCOLO, a single-arm phase II study, evaluated mirvetuximab soravtansine as third-line or later therapy in patients with FRα-high PSOC. Among 79 patients, the objective response rate was 51.9%, including six complete responses. Median duration of response was 8.3 months and median progression-free survival (PFS) was 6.9 months, supporting the use of mirvetuximab soravtansine after at least two prior platinum-based regimens in platinum-sensitive disease (9). Several further studies are ongoing. Study-420 is a phase II trial investigating mirvetuximab soravtansine combined with carboplatin followed by mirvetuximab soravtansine maintenance in patients with FRα-positive (≥25%) recurrent PSOC after one prior platinum-based treatment line (11). The MIROVA trial is a randomized, multicenter phase II study comparing mirvetuximab soravtansine plus carboplatin with standard platinum-based chemotherapy in patients with FRα-high disease who remain eligible for platinum therapy, with completion of enrollment anticipated in 2024 (10). The phase III GLORIOSA trial is evaluating mirvetuximab soravtansine plus bevacizumab as maintenance therapy in FRα-high PSOC following second-line platinum treatment (12). In addition, a phase II neoadjuvant study is assessing mirvetuximab soravtansine in the first-line perioperative setting, combining initial carboplatin with subsequent mirvetuximab soravtansine plus carboplatin. Another ongoing phase II study is exploring mirvetuximab soravtansine in combination with olaparib as maintenance therapy in patients with moderate FRα expression (≥50%) after platinum-based chemotherapy; BRCA-mutated patients must have previously received a PARP inhibitor. Although not FDA-approved in this setting, current NCCN guidelines and the PICCOLO study list mirvetuximab soravtansine as monotherapy for PSOC with high FRα expression (≥75%) and in combination with bevacizumab for tumors with moderate FRα expression (≥50%), based on the available clinical data (9, 13).

**Table 1** presents a descriptive overview of haematological adverse events reported across different trials and patient populations (carboplatin/gemcitabine in the AGO-OVAR 2.5 trial and the OCEAN trial; paclitaxel, doxorubicin, or topotecan in the control arm of the MIRASOL trial; carboplatin/PLD in the CALYPSO trial). As these trials differ in patient populations, treatment settings, and methodology, the data do not permit direct statistical comparison between regimens. With this limitation in mind, the reported rates of grade ≥3 thrombocytopenia, neutropenia, and anaemia appear numerically lower with mirvetuximab soravtansine than with the chemotherapy regimens shown, an observation that should be interpreted as hypothesis-generating rather than as evidence of a comparative safety advantage (14–16).

**Table 1:** Haematologic side effects (%) mentioned in the MIRASOL (Experimental and control arm), OCEAN-Trial, AGO-OVAR-2.5-Trial and Calypso-Trial (14–16).

,	*Mirvetuximab soravtansine* *MIRASOL-Trial*	*Paclitaxel, doxorubicin (PLD), topotecan* *Control Arm of MIRASOL-Trial*	*Carbo/gemzar* *OCEAN-Trial* *and AGO-OVAR-2.5-Trial*	*Carbo/PLD* *CALYPSO -Trial*
Thrombocytopenia *(any grade)*	8.0	Paclitaxel: 5.0 PLD: N/A Topotecan: 78.0	51.1	N/A
Thrombocytopenia *(grade ≥3)*	2.0	17.4	33.9	12.2
Neutropenia *(any grade)*	11.0	28.5	1.7	N/A
Neutropenia *(grade ≥3)*	0.9/2.0	17.4	21.9	27.5
Anemia *(any grade)*	9.6	34.3	58.0*	N/A
Anemia *(grade ≥3)*	0.9	10.1	20.0*	5

A recent case report by Njonou Noujiep et al. described the successful use of mirvetuximab soravtansine in a patient with FRα-positive PROC and concomitant PARP inhibitor-related MDS, achieving seven months of sustained disease control without worsening of the underlying hematologic disorder (17). In contrast, our patient had platinum-sensitive disease but could not undergo further platinum-based therapy because of severe hematologic toxicity and limited bone marrow reserve. This distinction is clinically relevant, as current evidence for mirvetuximab soravtansine is largely restricted to PROC.

Our patient received mirvetuximab soravtansine with PSOC after two prior lines of treatment. Due to her pre-existing haematological disease, our patient could not undergo platinum-based combination chemotherapy rechallenge. In our patient, mirvetuximab soravtansine has led to a pronounced radiological and serological partial response and a prolonged survival with low, but stable blood counts despite a known haematological malignancy, resulting in a PFS of nine months in a third recurrence of HGSOC. PFS was defined as the time from initiation of mirvetuximab soravtansine to radiologically confirmed disease progression. The long PFS (nine months) in our patient when compared to published reports of patients with a higher number of prior lines (median PFS 6.9 months) raises the hypothesis that earlier use of mirvetuximab soravtansine could be beneficial in patients with FRα-positive tumors — a question that would need to be addressed in larger, prospective studies. Further studies are needed to investigate the potential of mirvetuximab soravtansine in recurrent platinum-sensitive ovarian cancer with contraindication for combination-chemotherapy. Mirvetuximab soravtansine is approved by both FDA and EMA specifically for FRα-positive, platinum-resistant ovarian cancer (PROC) based on the MIRASOL trial, with EU authorization granted in November 2024 and full FDA approval following the same data. There is no approved indication in platinum-sensitive ovarian cancer (PSOC). This case suggests a potential role of mirvetuximab soravtansine for selected patients with platinum-sensitive but platinum-ineligible disease. Overall, mirvetuximab soravtansine appears to be a promising option for patients with PSOC and MDS due to its low haematotoxicity.

## Data Availability

The raw data supporting the conclusions of this article will be made available by the authors, without undue reservation.
